# Gender‐dependent multiple cross‐phenotype association of interferon lambda genetic variants with peripheral blood profiles in healthy individuals

**DOI:** 10.1002/mgg3.2292

**Published:** 2023-10-05

**Authors:** Debarati Guha Roy, Lucky Singh, Himanshu K. Chaturvedi, Sreedhar Chinnaswamy

**Affiliations:** ^1^ Infectious Disease Genetics National Institute of Biomedical Genomics Kalyani India; ^2^ Regional Centre for Biotechnology Faridabad India; ^3^ ICMR‐National Institute of Medical Statistics New Delhi India

**Keywords:** cross‐phenotype association, IFNL, IFNL3, IFNL4, interferon lambda, PheWAS, rs117648444, rs12979860, rs28416813, rs368234815, rs4803217

## Abstract

**Background:**

Type III interferons (IFN), also called as lambda IFNs (IFN‐λs), are antiviral and immunomodulatory cytokines that are evolutionarily important in humans. Given their central roles in innate immunity, they could be influencing other aspects of human biology. This study aimed to examine the association of genetic variants that control the expression and/or activity of IFN‐λ3 and IFN‐λ4 with multiple phenotypes in blood profiles of healthy individuals.

**Methods:**

In a cohort of about 550 self‐declared healthy individuals, after applying several exclusion criteria to determine their health status, we measured 30 blood parameters, including cellular, biochemical, and metabolic profiles. We genotyped them at rs12979860 and rs28416813 using competitive allele‐specific PCR assays and tested their association with the blood profiles under dominant and recessive models for the minor allele. IFN‐λ4 variants rs368234815 and rs117648444 were also genotyped or inferred.

**Results:**

We saw no association in the combined cohort under either of the models for any of the phenotypes. When we stratified the cohort based on gender, we saw a significant association only in males with monocyte (*p* = 1 × 10^−3^) and SGOT (*p* = 7 × 10^−3^) levels under the dominant model and with uric acid levels (*p* = 0.01) under the recessive model. When we tested the IFN‐λ4 activity modifying variant within groupings based on absence or presence of one or two copies of IFN‐λ4 and on different activity levels of IFN‐λ4, we found significant (*p* < 0.05) association with several phenotypes like monocyte, triglyceride, VLDL, ALP, and uric acid levels, only in males. All the above significant associations did not show any confounding when we tested for the same with up to ten different demographic and lifestyle variables.

**Conclusions:**

These results show that lambda interferons can have pleiotropic effects. However, gender seems to be an effect modifier, with males being more sensitive than females to the effect.

## Graphical abstract

The variance in phenotype data is not randomly distributed among groupings based on IFNL genetic variants and gender.

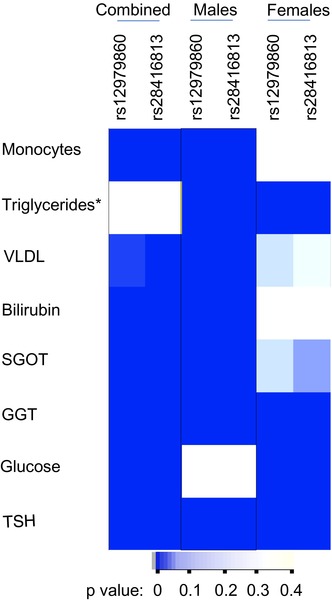

## 1 INTRODUCTION

Interferons (IFNs) are generally studied for their primary functions as antiviral molecules, and their roles in other aspects of human biology are less well explored. Since IFNs are indispensable cytokines not only during viral but also during other pathogen infections (Syedbasha & Egli, 2017) during the lifetime of an individual and since they have immunomodulatory functions (De et al., 2021, 2022), it is possible that their influence on the development of human physiology could be more pronounced than what we currently understand. The presence of genetic variants in the population that affect the biology of IFNs provides us with critical tools to examine this aspect at the epidemiological level.

**FIGURE 1:**
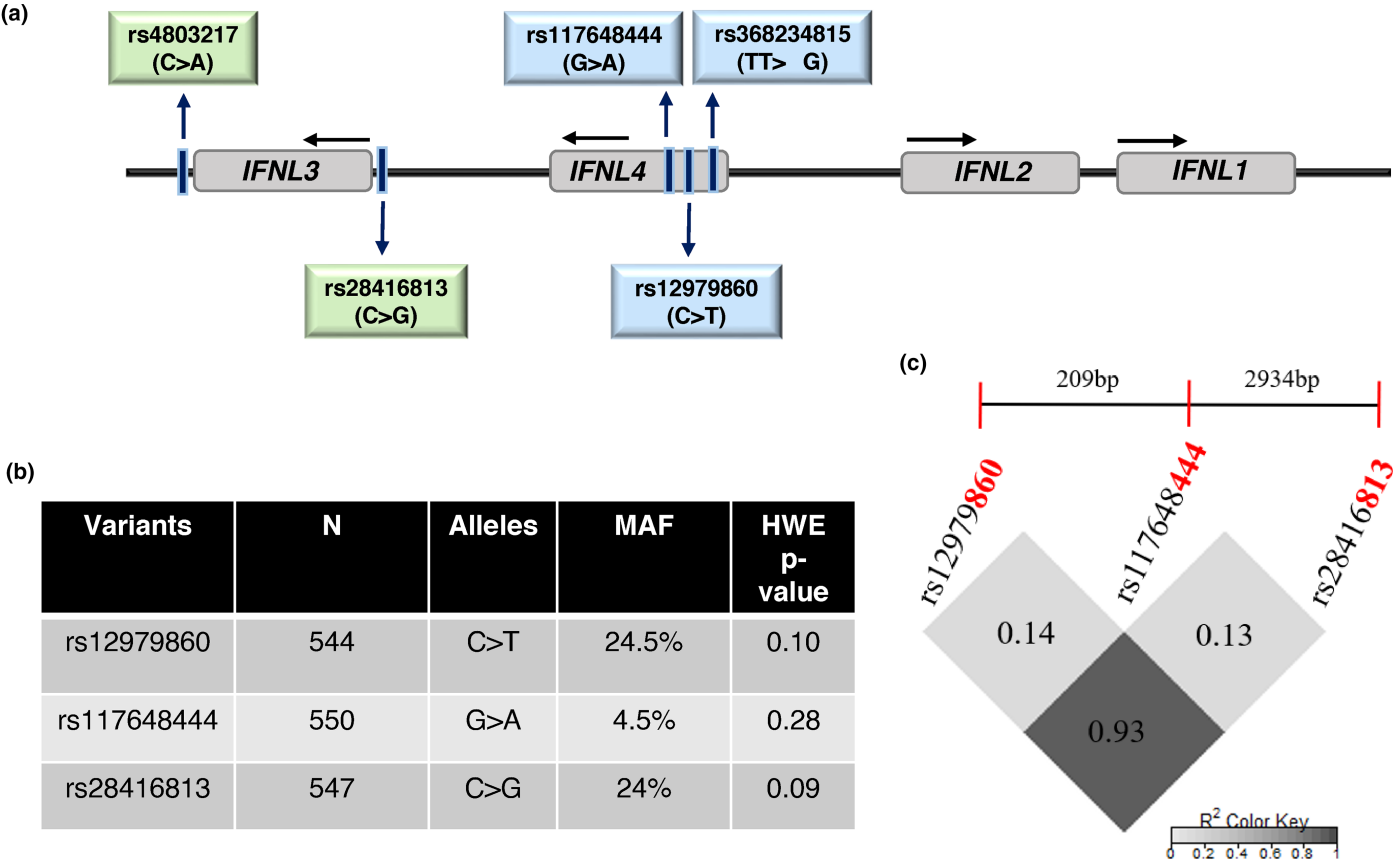
The human *IFNL* locus and their features in the study cohort. (a) Schematic of the human IFN lambda locus on chr. 19. The four genetic variants that control the expression and activity of IFN‐λ3 (rs4803217 and rs28416813) and IFN‐λ4 (rs368234815 and rs117648444) are shown along with the GWAS hit SNP rs12979860 (Ge et al., 2009). (b, c) The minor allele frequency (MAF) of the three variants, *p*‐value that shows their HWE status and their LD plot. The dinucleotide variant rs368234815 was in perfect LD with rs12979860 in line with our previous results (Bhushan et al., 2017) and hence its genotype status was inferred from the latter to determine the IFN‐λ4 status in a given individual along with the genotype information at rs117648444.

Interferon lambda (*IFNL* or IFN‐L or IFN‐λ) locus on human chromosome 19 consists of four duplicated genes; *IFNL1‐4* (Prokunina‐Olsson et al., 2013) (OMIM IDs 607,403, *IFNL3*, and 615,090, *IFNL4*). The first three *IFNL* genes were identified in 2003 after the draft human genome project was completed (Kotenko et al., 2003; Sheppard et al., 2003) the last IFN‐λ to be identified was IFN‐λ4, which was discovered in 2013 during follow‐up studies to hepatitis C virus (HCV) genome‐wide association studies (GWAS) (Prokunina‐Olsson et al., 2013). The *IFNL* locus has been under strong selective pressure during human evolution (Manry et al., 2011). Genetic variants have been identified that control the expression of IFN‐λ3 (rs28416813 and rs4803217) (Chinnaswamy et al., 2013; McFarland et al., 2014; Roy et al., 2021) and expression and activity of IFN‐λ4 (rs368234815 and rs117648444, respectively) (Prokunina‐Olsson et al., 2013; Terczyńska‐Dyla et al., 2014) (Figure 1a). These variants have been associated with several infectious and inflammatory diseases, including COVID‐19 (Chinnaswamy, 2016; Matic et al., 2023; Prokunina‐Olsson, 2019). The ∆G allele of the dinucleotide variant rs368234815 (TT/∆G) gives rise to an open reading frame that can translate a fully functional IFN‐λ4 protein (Figure 1a). The SNP (single nucleotide polymorphism) rs117648444 (G/A) is a non‐synonymous variant that changes the amino acid at 70th position in IFN‐λ4 from proline to serine (P70 or S70); the S70 variant has significantly reduced activity in terms of levels of IFN‐stimulated gene (ISG) expression compared to P70 in vitro and it phenocopies the TT allele that abolishes the expression of an active IFN‐λ4 due to disruption of the ORF, in association with tests involving HCV treatment response cohorts (Bhushan et al., 2017; Terczyńska‐Dyla et al., 2014). The SNPs rs28416813 (C/G) and rs4803217 (C/A) present in the 5′UTR and 3′UTR, respectively, of the *IFNL3* gene potentially regulate the expression levels of IFN‐λ3 (Chinnaswamy et al., 2013; McFarland et al., 2014; Roy et al., 2021). The SNP rs12979860 (C/T) was the strongest signal in the HCV GWAS and is usually included as a proxy SNP for *IFNL* gene functional variants (Ge et al., 2009; Prokunina‐Olsson, 2019).

Both IFN‐λ3 and IFN‐λ4 are strong antiviral cytokines in vitro and they can also modulate immune cell functions (De et al., 2021; De et al., 2022; Hamming et al., 2013; Lauber et al., 2015; Obajemu et al., 2017). However, no studies have been conducted to examine their influence on the physiology of healthy individuals. There have been limited studies that have reported on the allele frequencies of the *IFNL* SNPs from the resident Indian population. To fulfill the above lacunae, we undertook this study and report some interesting observations from it.

## 2 MATERIALS AND METHODS

### 2.1 Ethical compliance, study population, and blood tests

The study was approved by the National Institute of Biomedical Genomics committee for ethics on human studies (certificate No. NIBMG/2022/1/0018). The study was based in the city of Kalyani, a small township in Nadia district of West Bengal state in India. The participants were included based on a questionnaire that had several questions on demographics, lifestyle, socioeconomic status, health, and disease aspects. Individuals were excluded if they were pregnant and had any of the chronic diseases like diabetes, hypertension, anemia, etc.; they were excluded if they had diseases of thyroid, kidney, liver, heart or were suffering from any gastrointestinal, cardiovascular, neurological, gynecological, respiratory (asthma), urinary, endocrine (thyroid), skin disorders or were taking any long‐term medications or had any chronic infectious diseases like HCV, hepatitis B virus, human immunodeficiency virus infections, tuberculosis, malaria, leishmaniasis, and cancer. Individuals who suffered from dengue, chikungunya, Japanese encephalitis, swine flu, chicken pox, cholera, typhoid, COVID‐19 in the past 6 months or had a blood transfusion in the past 6 months were excluded. Individuals were selected only if they had not suffered from an acute disease episode like fever/cold/cough in the previous 30 days.

After obtaining informed consent, 6 mL of their venous blood was drawn in EDTA vials, EDTA + sodium fluoride vials, and in plain vials by a trained phlebotomist, and 5 mL of the blood was sent to a commercial lab that was approved by the national accreditation board for testing and calibration of laboratories (NABL) of the Indian government. Thirty quantitative phenotypes (shown in Table S1 and S2) were measured that included biochemistry, hematology, and metabolic panels routinely carried out by the lab for regular health check‐up of its customers.

### 2.2 Genotyping

We selected four *IFNL* genetic variants for the study: rs368234815, rs12979860, rs117648444, and rs28416813 (Figure 1a). Genomic DNA was isolated from blood using the QIAamp DNA mini kit (Qiagen). The concentration and quality of the isolated genomic DNA was measured using absorbance spectroscopy (Nanodrop, ThermoFisher). The genotyping of all the samples were carried out using competitive allele‐specific polymerase chain reaction (KASP, LGC Genomics, UK) method for the SNPs rs12979860, rs117648444, and rs28416813. The assay was carried out in Quantstudio 5 (Applied Biosystems) real‐time PCR machine. For genotyping of rs368234815, a subset of seventy‐seven samples were sequenced using Sanger sequencing from an amplicon generated from the primers: (1) IL28B4kbxhoRev: 5’‐GATATCCTC GAGCCCGGATTTCAGGAC‐3′ and (2) IL28B4kbKpnFor: 5’‐GATATCGGTACCGCCCTGGACGGGAAAG‐3′. The Genbank reference sequence numbers for: *IFNL3* is NM_001346937.2 and for *IFNL4* is NM_001276254.2.

### 2.3 Statistical tests and data analysis

Hardy–Weinberg equilibrium (HWE) was tested using Plink v1.90b6.17 (Purcell et al., 2007). Linkage disequilibrium (LD) was calculated in RStudio 4.1.0 (RStudio Team, 2021) using LDheatmap package. LD plot was also made using RStudio 4.1.0. Dominant or recessive models for the minor alleles were selected, as they have been routinely used in HCV studies (Chinnaswamy, 2016). The normality of the data was checked by using the Kolmogorov–Smirnov test. The student *t*‐test and Wilcoxon Mann–Witney U test were used for comparing any two groups for significant differences in the midpoints of the data. The F‐test was used for comparing variances of any two groups. The Chi‐square test was used to check the association between genetic variants and demographic variables. Multiple linear regression was used to test the effect of genetic variants on blood phenotypes in the presence of other demographic and lifestyle variables, including age and/or gender. For all tests, *p* < 0.05 was considered as significant, unless specified; further, no multiple testing correction was carried out on the reported *p*‐values except where specified. Data from the UK biobank was accessed through: Global Biobank Engine (GBE), Stanford, CA (URL: http://gbe.stanford.edu) [June–July 2023] (McInnes et al., 2019). To compare the effect size of SNPs on monocyte counts and SGOT levels between our study cohort and that from the GBE, we calculated the standardized (scaled) β coefficient from our data specific to males using multivariate linear regression with age as a covariate. All the statistical tests were performed in RStudio 4.1.0. The violin plots were made using Graphpad Prism 8.

## 3 RESULTS

### 3.1 Study cohort and IFNL genetic variants

While selecting the participants for the study, we applied strict exclusion criteria to rule out any chronic diseases or any viral diseases in the previous 6 months, since viral infections can induce IFNs and could potentially influence the results. We were able to select about 552 self‐declared healthy individuals to be included in the study. The age of the participants ranged from 11 to 94 years (median 32 years) with similar distribution in both the genders. The cohort had 268 females and 284 males. In general, the cohort appeared to be healthy based on the dispersion of data within the normal range in most of the 30 blood parameters that we measured (Table S1). However, there were some exceptions: among other minor deviations, the erythrocyte sedimentation rates, bilirubin (direct), and SGOT levels were higher than normal in both genders; triglycerides and SGPT levels were higher and creatinine levels were lower only in males while ALP levels were higher only in females. Several phenotypes showed highly significant differences between males and females, some were on expected lines (as per the cut‐off criteria set by the lab where the tests were carried out), but there were up to 16 phenotypes where a significant difference was seen that was not expected (Table S2).

We genotyped all SNPs except rs368234815 by using the KASP assays which are based on competitive allele‐specific PCR (LGC Genomics, UK). All of the genotyped SNPs were in HWE (*p* > 0.05) (Figure 1b). We genotyped rs368234815 in only a subset of individuals (*n* = 77) using Sanger sequencing and, in line with our previously published results (Bhushan et al., 2017) from a small HCV cohort, we found that it was in perfect LD with rs12979860 (LD, *r* ^2^ = 1). Therefore, we used rs12979860 as a proxy for rs368234815 in the rest of the study. The minor allele frequencies (MAF) and HWE *p*‐values for rs12979860, rs117648444, and rs28416813 are shown in Figure 1b. The SNPs rs12979860 and rs28416813 are in strong LD with each other (Figure 1c), and they were used under the dominant or recessive models to test for association with multiple phenotypes.

### 3.2 Distribution of variance between the groups within dominant and recessive models of IFNL genetic variants in the combined cohort or in male and female sub‐cohorts

**FIGURE 2:**
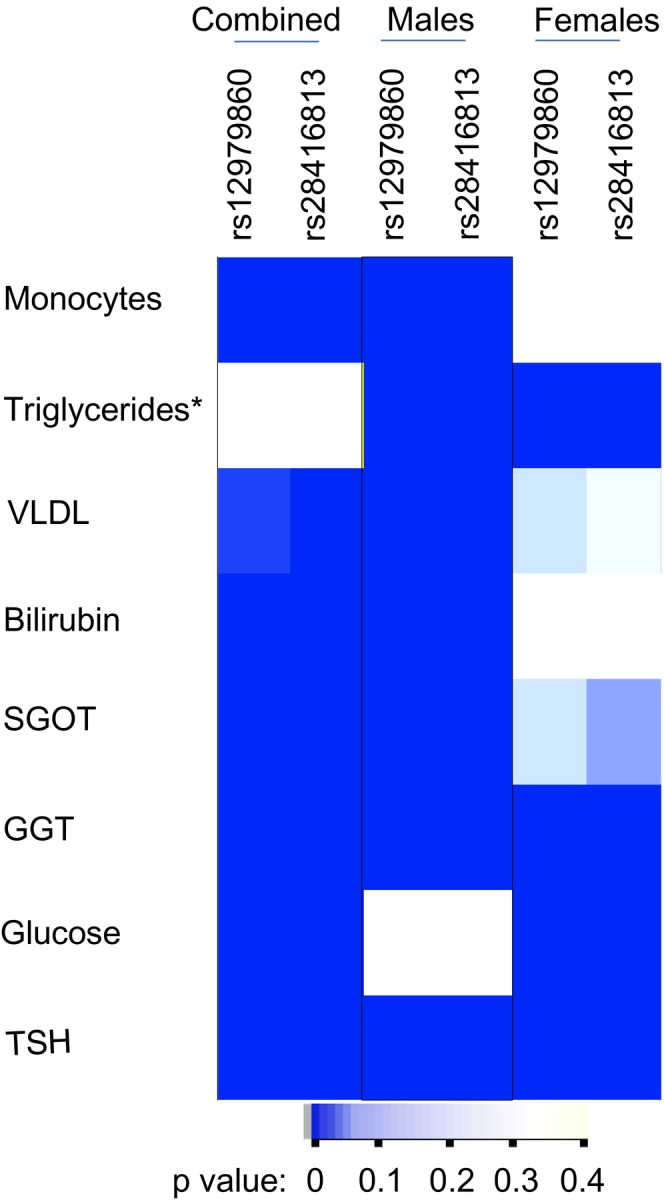
Non‐random distribution of variance according to *IFNL* SNPs and gender in multiple phenotypes in the study cohort. Heat map shows the *p*‐value distribution in the combined cohort and the gender sub‐cohorts after applying Bonferroni correction for multiple testing as described in the Results section; the darker color is suggestive of highly significant association of the variance between the major homozygotes group and the group with the remaining individuals (dominant model for the minor allele). The *p*‐values were derived from the F‐statistic. The results from only the dominant model are shown here. *Triglyceride levels qualified for the criteria by showing significant *p*‐values for both the SNPs under the recessive model even though the dominant model showed insignificant *p*‐values in the combined cohort; however, they showed highly significant *p*‐values for both the SNPs under the dominant model only when the cohort was stratified on gender and interestingly the association was in opposite directions in males vs. females. Abbreviations: VLDL‐cholesterol: Very Low‐Density Lipoprotein‐cholesterol, SGOT: Serum Glutamic Oxaloacetic Transaminase, GGT: Gamma Glutamyl Transferase, TSH: Thyroid Stimulating Hormone.

Our study design is loosely based on the concept of Mendelian Randomization (Adam, 2019), wherein random segregation of alleles occurs during meiosis and that any association that the alleles may show with any phenotype(s) in the offspring should be free from confounding. We tested if the variance that is naturally present in the data that we collected for the 30 phenotypes had a random distribution between the two groups formed under either the dominant or recessive models for the IFNL genetic variants. We saw some highly significant associations in many of the phenotypes with *p*‐values ranging from 5 × 10^−2^ to 3 × 10^−35^ (not shown). In order to filter out the strongest associations, we set the following criteria: (a) a *p*‐value <4.1 × 10^−4^ (after Bonferroni correction for 120 tests: two SNPs under two models for 30 phenotypes); (b) if it is a dominant model, at least one of the two SNPs should have a significant *p*‐value; and (c) if it is a recessive model, both SNPs should show a significant *p*‐value for association. We saw that eight phenotypes were able to survive these stringent criteria and are shown in Figure 2. Next, we tested if the phenotype variance we saw between the groups was randomly distributed between the males and females (Figure 2). We chose only the dominant model to compare the distribution among the two genders as the sample numbers within the gender sub‐cohorts were more than 30 under this model in order to justify the use of a parametric test like F‐test (Ghasemi & Zahediasl, 2012). The variance distribution between the genotype groups under the dominant model was more significantly different in males compared to the females in many of the phenotypes except in blood glucose levels, where females contributed to the bulk of the association that we saw in the combined cohort. In some phenotypes like GGT and TSH, both males and females contributed to the significant association seen in the combined cohort, whereas in triglycerides, the association in males and females was in opposite directions (Figure 2). These results suggest to us that the *IFNL* genetic variants could potentially influence multiple phenotypes in humans in a gender‐specific manner.

### 3.3 Significant associations between multiple phenotypes and IFNL genetic variants

Since the data for all the phenotypes showed a non‐normal distribution (Kolmogorov–Smirnov test; *p*‐value <1.1 × 10^‐8^), we used a non‐parametric test (Wilcoxon Mann–Witney U test) to look for significant differences in the midpoints of the quantitative traits in each of the groups being compared. We found no association with any of the 30 phenotypes with either of the SNPs rs12979860 or rs28416813 using either the dominant or the recessive models (Table 1; data not shown for all phenotypes). It is known from several previous studies, including ours (Astudillo et al., 2020; Chinnaswamy et al., 2017; Eslam et al., 2015), that gender can be an effect modifier with *IFNL* genetic variants. Therefore, we stratified the cohort into males and females and tested all the parameters under the two models and also based on IFN‐λ4 copy number and activity status (Table 1; data shown only for significant associations; a total of 270 tests were carried out: 120 for the two SNPs under two models for 30 phenotypes; a total of 150 tests for five comparisons of IFN‐λ4 copy number and activity status with 30 phenotypes). There were several significant associations (*p* < 0.05) that we observed in males (a total of 15 out of a total of 270 tests conducted) and a few in females (a total of 5 out of a total of 270 tests conducted). The associations that were significant (*p* < 0.05) were not randomly distributed across males and females (*p* = 0.02; Chi‐square test). This suggests that there is a gender‐specific effect of the *IFNL* genetic variants on up to 11 phenotypes (Table 1).

**TABLE 1:** Association of blood profile phenotypes with *IFNL3/4* polymorphisms.

	Combined cohort				Male sub‐cohort				Female sub‐cohort			
	rs12979860 (C>T)		rs28416813 (C>G)		rs12979860 (C>T)		rs28416813 (C>G)		rs12979860 (C>T)		rs28416813 (C>G)	
	*N* = 544		*N* = 547		*N* = 278		*N* = 281		*N* = 266		*N* = 266	
	C/C = 317		C/C = 323		C/C = 161		C/C = 163		C/C=156		C/C = 160	
	C/T = 187		C/G = 185		C/T = 96		C/G = 99		C/T = 91		C/G = 86	
	T/T = 40		G/G = 39		T/T = 21		G/G = 19		T/T = 19		G/G = 20	
	Dom	Rec	Dom	Rec	Dom	Rec	Dom	Rec	Dom	Rec	Dom	Rec
Platelet count	0.66	0.09	0.43	0.25	0.80	0.85	0.70	0.96	0.37	**0.02**	0.12	0.14
% of Monocytes	0.14	0.29	0.14	0.94	**0.007**	0.11	**0.01**	0.24	0.50	0.87	0.76	0.24
SGOT	0.21	0.49	0.09	0.90	**0.02**	0.05	**0.01**	0.09	0.65	0.32	0.94	0.10
Albumin	0.22	0.20	0.34	0.29	0.31	0.84	0.50	0.73	0.35	**0.01**	0.32	**0.04**
Globulin	0.98	0.68	0.62	0.83	0.38	**0.03**	0.50	0.12	0.43	0.13	0.16	0.33
Uric Acid	0.81	0.27	0.86	0.10	0.86	**0.01**	0.77	**0.02**	0.59	0.90	0.32	0.67

	Combined cohort					Male sub‐cohort					Female sub‐cohort				
	P70_1 vs S70*	P70_1 vs No‐IFNL4	P70_2 vs S70*	P70_2 vs No‐IFNL4	P70_1 vs P70_2	P70_1 vs S70*	P70_1 vs No‐IFNL4	P70_2 vs S70*	P70_2 vs No‐IFNL4	P70_1 vs P70_2	P70_1 vs S70*	P70_1 vs No‐IFNL4	P70_2 vs S70*	P70_2 vs No‐IFNL4	P70_1 vs P70_2
% of Eosinophils	**0.02**	0.45	0.26	0.55	0.38	0.47	0.88	0.51	0.93	0.96	**0.01**	0.20	0.39	0.43	0.18
% of Monocytes	0.61	0.27	0.63	0.71	0.85	0.78	**0.03**	1	0.28	0.84	0.53	0.56	0.47	0.45	0.66
Triglycerides	0.75	0.92	**0.03**	**0.01**	**0.01**	0.64	0.80	0.13	**0.01**	**0.01**	0.20	0.97	0.15	0.49	0.44
VLDL	0.75	0.96	**0.03**	**0.01**	**0.01**	0.64	0.80	0.13	**0.01**	**0.01**	0.20	0.95	0.15	0.50	0.43
ALP	**0.01**	0.08	0.13	0.43	0.82	**0.01**	0.05	0.10	0.28	0.75	0.70	0.58	0.77	0.89	0.98
Creatinine	0.22	0.36	0.56	0.94	0.61	0.94	0.73	0.28	0.16	0.26	0.05	0.19	**0.04**	0.17	0.67
Uric Acid	0.63	0.85	0.44	0.25	0.23	0.35	0.26	0.11	**0.03**	**0.01**	0.95	0.65	0.93	0.97	0.85

*Note*: Only significantly associated (*p*<0.05) phenotypes out of the 30 phenotypes in either the male or female sub‐cohort are shown here. A total of 270 tests were carried out: 120 for the two SNPs under two models for 30 phenotypes; a total of 150 tests for five comparisons of IFN‐λ4 copy and activity status with 30 phenotypes. All the *p*‐values shown are from Wilcoxon Mann Whitney U‐test. Dominant (Dom) and recessive (Rec) models for the minor alleles of each SNP (e.g., C/C vs C/T + T/T for rs12979860 dominant model, C/C + C/T vs T/T for rs12979860 recessive model) and IFN‐L4 status (No IFNL4 or 1 copy of IFNL4‐P70 or 2 copies of IFNL4‐P70, and 1 copy of IFNL4‐S70*) were used for the comparisons. *p*‐values <0.05 were considered significant and highlighted in bold. VLDL‐cholesterol: Very Low‐Density Lipoprotein‐cholesterol, SGOT: Serum Glutamic Oxaloacetic Transaminase, ALP: Alkaline Phosphatase

* The IFNL4‐S70 group has some IFNL4‐P70 alleles as heterozygotes along with IFNL4‐S70 alleles; the combined cohort has 44 copies of IFNL4‐S70 alleles and 10 copies of IFNL4‐P70 alleles, the males sub‐cohort has 24 IFNL4‐S70 alleles and 7 copies of IFNL4‐P70 alleles, while the female sub‐cohort has 20 IFNL4‐S70 alleles and 3 copies of IFNL4‐P70 alleles.

Next, we tested if several demographic and lifestyle parameters that we collected as part of the metadata could be potential confounders in our significant results shown for the 11 phenotypes in Table 1. We had collected a total of 11 demographic and lifestyle factors in our metadata (Table S3). For males and females separately, we tested if any of these factors significantly associated with the two SNPs rs12979860 and rs28416813 based on the groupings under dominant or recessive models and also groupings based on IFN‐λ4 copy number and activity status using Chi‐square tests. None of the demographic/lifestyle factors showed a significant association (*p* < 0.05) with either of the SNP or based on IFN‐λ4 copy number and activity status in males (data not shown); however, in females, we saw three factors (housing type, annual income, and source of drinking water) showing a significant association (*p* < 0.05) with either of the SNPs or with the IFN‐λ4 copy number and activity status (Table 2). We deemed these three variables to be potential confounders in the significant association we saw in females with the *IFNL* variants highlighted in Table 1. Out of the four phenotypes that showed significant association with *IFNL* variants in females (shown in Table 1), two, i.e., platelet counts and albumin levels also significantly (*p* < 0.05) associated with at least one of the three demographic/lifestyle variables (Table 2), suggesting that the latter could be confounders in our results on significant association between *IFNL* genetic variants and the two phenotypes.

**TABLE 2:** Association of the demographic and lifestyle variables with the dominant and recessive models of *IFNL3/4* SNPs and with the *IFNL4* copy number and activity status in females.

	rs12979860		rs28416813		IFNL4 Status			
	Dominant	Recessive	Dominant	Recessive	No‐IFNL4	P70_1	P70_2	S70*
Type of housing (significance between Pukka house, Semi‐pukka and Kucha house)	0.6937	0.1479	0.711	0.3839	**0.007533**			
Annual income of family (significance between: Rs. <25000–1 lakhs; 25000 to 1.5 Lakhs; 1.5 Lakhs to 5 Lakhs and >5 Lakhs)	0.7563	**0.02176**	0.6249	**0.001377**	**0.02139**			
Source of drinking water in the household (significance between Tap water; Hand pump and tube well)	**0.02755**	0.4872	0.1067	0.3843	**0.00378**			

		Platelet count	% of Eosinophils	Albumin	Creatinine
Type of House (pukka house‐1, Semi‐pukka‐2, Kucha‐3)	**1 vs 2**	**0.02233**	0.2059	0.2302	0.8037
	**1 vs 3**	0.1045	0.9582	0.2941	0.8727
	**2 vs 3**	**0.006799**	0.4145	0.8969	0.826
Annual income of family (<25000‐1 Lakhs‐1; 25000 to 1.5 Lakhs‐2; 1.5 Lakhs to 5 lakhs‐3; >5 Lakhs‐4)	**1 vs 2**	0.1522	0.1598	0.715	0.8333
	**1 vs 3**	0.7347	0.2825	0.8076	0.8904
	**1 vs 4**	0.2567	0.4847	0.3249	0.5304
	**2 vs 3**	0.1794	0.9229	0.9505	0.6514
	**2 vs 4**	0.7428	0.953	0.09911	0.2369
	**3 vs 4**	0.3083	1	0.1714	0.3438
source of drinking water in the household (Tap water‐1, Hand pump‐2, tubewell‐3)	**1 vs 2**	0.4656	0.9398	0.498	0.804
	**1 vs 3**	0.1408	0.8094	**0.0133**	0.7374
	**2 vs 3**	0.9654	0.8987	0.2913	0.6124

*Note*: This table has data from only the female sub‐cohort as we did not see any significant association using the Chi‐square test between the genetic variants and any of the demographic and lifestyle variables in males. In females, we found a significant association (*p* < 0.05) between three demographic/lifestyle factors shown in this table and the two SNPs (tested under two models) and on *IFNL4* status. All the *p*‐values shown in the first part of the table are from the Chi‐square test. The four phenotypes in females that showed a significant association with IFNL genetic variants (shown in Table 1) were also tested for association with the three demographic/lifestyle variables using Mann Whitney U test and significant differences are highlighted in bold as shown in the second part of the table. *p*‐values < 0.05 are considered as significant. S70*‐as explained for Table 1.

**FIGURE 3:**
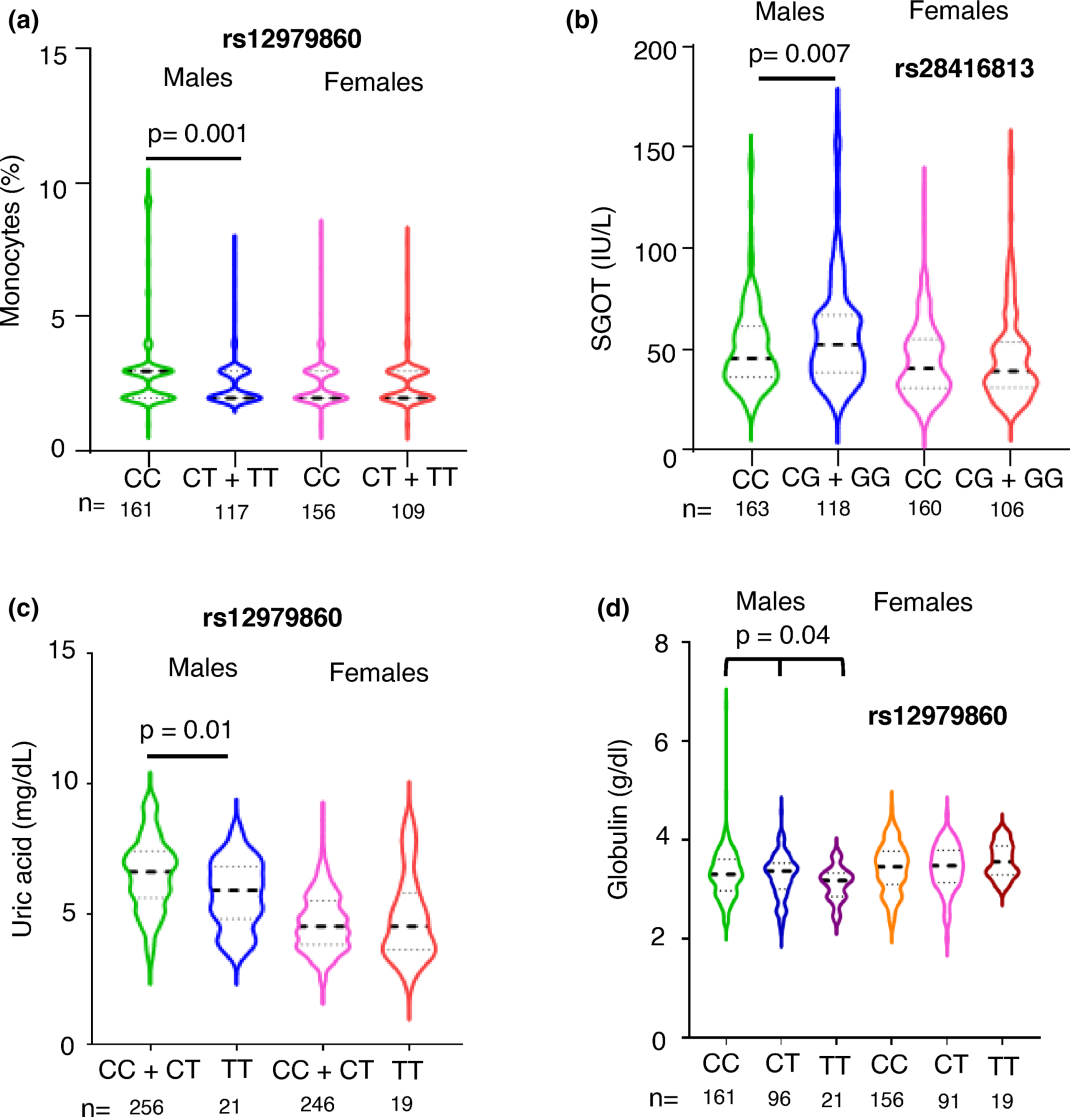
Multiple phenotypes significantly associate with *IFNL* SNPs only in males. (a–d) Violin plots showing the distribution of data around the median (thick dashed line shows median and the two thin lines separate the quartiles in each half) for the phenotypes shown according to the *IFNL* SNPs under the dominant or recessive models (for a, b and c) or additive model (for d) for the minor allele as shown. All the *p*‐values shown were for the effect of the individual SNPs derived from multiple linear regression analysis with age as the covariate (fully described in Table S4). No significant effect of the SNPs was seen in the female sub‐cohort.

**FIGURE 4:**
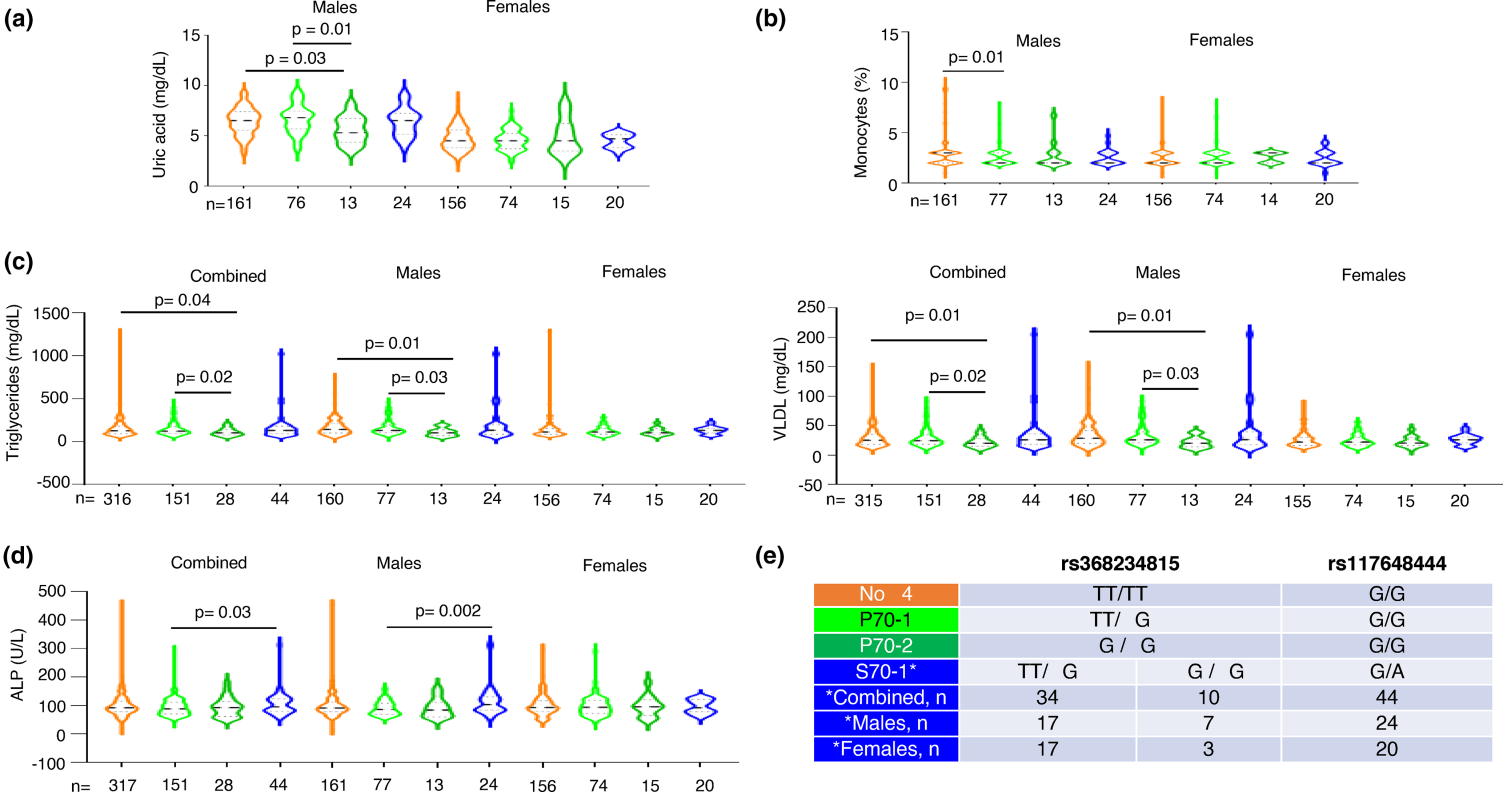
Multiple phenotypes significantly associate with groupings based on IFN‐λ4 copy number and status. (a–d) The IFN‐λ4 status was inferred from the genotype information at rs12979860 and rs117648444 for each individual before grouping them in to one of the four groups‐ no‐IFN‐λ4, P70‐1 (having a single copy of the high activity P70 variant of IFN‐λ4), P70‐2 (having two copies of the high activity P70 variant of IFN‐λ4) and S70‐1 (while the large majority of the individuals in this group had one copy of the low‐activity S70 variant of IFN‐λ4, a minority of them had diplotypes, i.e. had one copy of the high activity P70 variant as well, as explained in e). (e) The IFN‐λ4 status based on copy number and activity level explained. A minority (10 in the combined, 07 in the males and 03 in the female sub‐cohort) of individuals had both a high activity and low‐activity IFN‐λ4 variant in them; this grouping strategy allowed us to have more power in testing the S70 variant due to the low MAF of rs117648444 SNP (see Figure 1b). All the *p*‐values shown were for the effect of the individual SNPs/variants derived from multiple linear regression analysis with age and gender (for the combined cohort) or age only (for the individual gender sub‐cohorts) as the covariate(s) (fully described in Table S4).

Only those significant associations between *IFNL* genetic variants and peripheral blood phenotypes that were free from confounding by the 11 demographic/lifestyle factors (as shown in Table S3) were chosen and adjusted for age using multiple linear regression. We saw that the only two significant associations we had seen in females, i.e., with eosinophil % and creatinine levels became insignificant after adjusting for age (Table S4; *p*‐values only for the genetic variants are shown in the table). The remaining significantly associated phenotypes in males are shown as violin plots in Figures 3 and 4. Monocytes and SGOT levels showed significant association after adjusting for age in males under the dominant model, while uric acid levels were significantly associated under the recessive model (Figure 3a–c).

While the recessive model did not show any significant association between rs12979860 and globulin levels (*p* = 0.05), an additive model showed a significant association after adjusting for age (Figure 3d). The additive models for the other three phenotypes shown in Figure 3 did not improve on the significance level (data not shown) showed by dominant or recessive models tested, supporting our assumption that the latter models have better power in detecting a significant association with IFNL genetic variants.

When we tested for absence or presence of one or two copies of P70 IFN‐λ4 (P70‐1 or P70‐2) or one copy of S70 IFN‐λ4 (S70‐1) (there was only one individual with two copies of S70 variant allele and was excluded from the analysis and there were ten individuals in the combined cohort, seven individuals in the males and three in the females sub‐cohort that were also homozygous for the ΔG allele at rs368234815 and heterozygous at the rs117648444 position and hence had both S70 and P70 variant copies in them as shown in Figure 4e; this strategy was adopted to have better power in the analysis involving the S70 group as the sample sizes with only one copy of S70 variant was low due to the lower frequency of the minor allele in our population) based on the genotype data at SNPs rs12979860 (inferred for rs368234815) and rs117648444, we saw that P70‐2 carrying males had significantly lower uric acid levels than no‐IFN‐λ4 or P70‐1‐carrying individuals (Figure 4a). However, with monocyte % levels, P70‐1 showed a significant association with the no‐IFN‐λ4 genotype (Figure 4b). With both Triglyceride and VLDL, having two copies of P70 proved beneficial in maintaining significantly lower levels of the lipids when compared to having no‐IFN‐λ4 or one copy of P70 (Figure 4c); even though the combined group had significant *p*‐values, they seem to be driven by the male sub‐cohort as females did not show such an association. In both these lipid profiles or in monocyte or uric acid levels, the S70 variant does not seem to have any significant association with the other groups (Figure 4a‐c); however, in ALP levels, the low‐activity S70 variant showed significant association with the P70 variant (one copy) in the combined group and the association further strengthened only in the male sub‐cohort (Figure 4d). Previous studies have used a different strategy to examine the effect of the weaker S70 variant compared to the stronger P70 variant (Gadalla et al., 2020; Terczyńska‐Dyla et al., 2014). They have compared three groupings instead of the four groupings we have adopted in Figure 4. The P70 variants: both one and two copies, including some one copy S70 variants are placed in a single group and compared against no‐IFN‐λ4 carriers and those that carry only one copy of S70 variant (Terczyńska‐Dyla et al., 2014). We too tried this strategy and tested all the five phenotypes shown in Figure 4 and found that except for monocyte levels (%), in the comparison with no‐IFN‐λ4 vs. P70, no other comparison either in the combined or males cohort showed a significant association after adjusting for age and gender (Table S5). Our strategy therefore has captured the dosage effect of the P70 variant (in case of triglycerides and VLDL levels, Figure 4c) and also the weak vs strong IFN‐λ4 effect (ALP in Figure 4d) among others, better than this alternate strategy adopted in previous reports (Gadalla et al., 2020; Terczyńska‐Dyla et al., 2014) (Figure 4 and Table S5). The discrepancies in results from the two strategies could be related to issues of sample sizes (especially of the S70 carriers) and therefore, statistical power and/or different nature and size of the effects of the weak vs. strong IFN‐λ4 variants.

### 3.4 Predictor variables of monocyte, SGOT, uric acid, and globulin levels in males using multiple linear regression analysis

We used the significant associations we saw in males for either of the SNPs rs12979860 and rs28416813 with the four phenotypes: monocyte, SGOT, uric acid, and globulin levels (Table 1 and Figure 3), to build a linear regression model to predict the phenotype levels using the 11 different demographic/lifestyle factors shown in Table S3 as covariates (Table 3). Only data from two phenotypes, i.e., monocyte and uric acid levels, were able to yield a linear regression model that had significance based on F‐statistic *p*‐value (Table 3). However, even in these two phenotypes, the variation seen in the dependent variable that was explained by all the independent variables was =/<10%, suggesting the inefficiency of the model. Nonetheless, the *IFNL* genetic variants remained significant in their effect on both monocyte and uric acid levels in this multiple regression model. The SNP rs28416813 also had a significant effect on predicting SGOT levels in the multiple regression model, even though the model itself was insignificant.

**TABLE 3:** The effect of *IFNL3/4* SNP on blood phenotypes in presence of other demographic/lifestyle variables in males.

Dependent variable	Independent variable	Coefficient estimates	*p*‐value	*R* ^2^ value	*p*‐value of F statistics
Monocyte %	rs12979860 (Dominant model)	−0.50131	**0.01074**	0.1051	**0.002827**
	Place of house	−0.64153	**0.00836**		
	Annual income of family	−0.3111	**0.0468**		
SGOT	rs28416813 (Dominant model)	6.3628	**0.0324**	0.05467	0.2231
Uric acid	rs12979860 (Recessive model)	−0.82105	**0.0101**	0.08942	**0.01394**
	Annual income of family	0.2671	**0.0477**		
Globulin	rs12979860 (Recessive model)	−0.21317	0.0551	0.07001	0.07515
	Age group	0.11468	**0.0056**		

*Note*: Multiple linear regression was performed to regress the data of the dependent variables (phenotypes as shown) using the genetic variants along with 11 demographic/lifestyle factors as independent variables. Only those independent variables that showed a significant effect in the model are shown in this table. *p*‐value < 0.05 was considered as significant and highlighted in bold.

### 3.5 IFNL genetic variants in a global genotype–phenotype database

**FIGURE 5:**
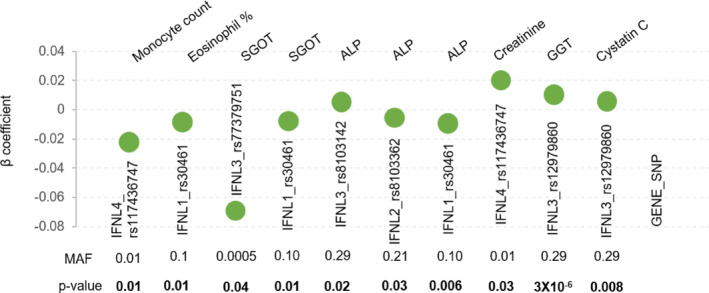
Summary statistics of PheWAS data derived from the UK biobank showing the multiple cross‐phenotype association of *IFNL* SNPs. The data was accessed from Global Biobank Engine (GBE), Stanford, CA (URL: http://gbe.stanford.edu) [June–July 2023]. MAF‐minor allele frequency.

The simultaneous availability of genome‐wide genotype information from GWAS and large number of phenotypes in health cohorts have augmented genotype–phenotype correlation studies that have been carried out as phenome‐wide association studies (pheWAS) and the summary statistics are made available in public databases. The GBE (McInnes et al., 2019) that has PheWAS data from the UK biobank is one such resource. We searched the GBE for all the phenotypes that associated with any of the known *IFNL* genetic variants (Figure 5). We saw that the *IFNL* genetic variants with varying MAFs were associated with multiple phenotypes similar to our results. While the *p*‐values are comparable, the significant association in our study was only from the male sub‐cohort, while the gender‐stratified data in the GBE was not accessible.

**FIGURE 6:**
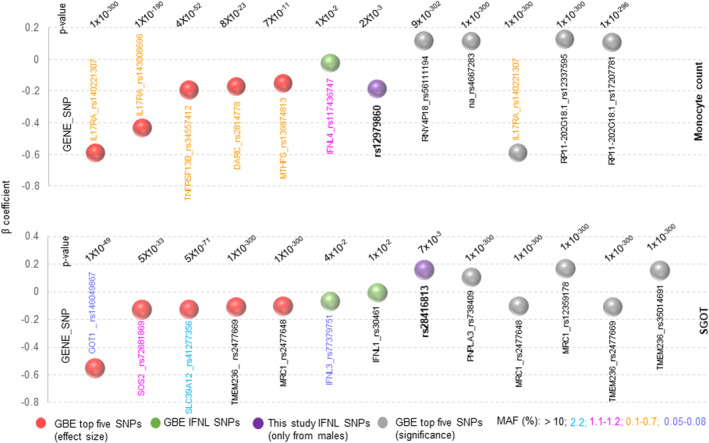
The effect sizes of *IFNL* SNPs from our study for male‐specific monocyte counts and SGOT levels are better than that for many of the top genes/SNPs for the respective phenotypes represented in the GBE. The summary statistics (beta values, *p*‐values and MAFs) for the top genes/SNPs in terms of effect sizes (red balls) or significance (gray balls) were obtained from the GBE PheWAS database. The MAFs are represented by showing the gene/SNP IDs in different colors, as explained at the bottom of the figure. Note that the majority of the genes/SNPs that have the best effect and significance on the two phenotypes have low MAFs. The beta coefficient for the SNPs derived from our study (only from male sub‐cohort), shown as purple balls, were calculated in a multiple linear regression model after standardization with age as a covariate. GBE, global biobank engine; MAF, minor allele frequency.

We next looked at the topmost genes/SNPs that are associated with monocyte counts or SGOT levels from the GBE (Figure 6) and compared the beta coefficients with that of *IFNL* genetic variants within the database and to those from our study. We compared the top five genes/SNPs available for the two phenotypes in the GBE for effect size and for significance (*p*‐value), with the *IFNL* SNPs. We saw that even though the GBE *IFNL* SNPs had moderate effect sizes and low MAFs, the SNPs rs12979860 for monocyte counts and rs28416813 for SGOT levels from our male sub‐cohort had a scaled beta coefficient value that was better than at least six of the top ten genes/SNPs for each of the phenotypes available in the GBE.

## 4 DISCUSSION

The *IFNL* genetic variants have lately been associated with several infectious and inflammatory diseases in humans (Chinnaswamy, 2016; Prokunina‐Olsson, 2019), even though they gained initial recognition for their association with post‐therapy HCV clearance in GWAS (Ge et al., 2009; Suppiah et al., 2009; Tanaka et al., 2009). In this study, we show that they also associate with several health phenotypes measured in peripheral blood of healthy individuals.

The observed effects could be due to differential regulation of *IFNL3* or *IFNL4* gene expression. Our methodology is not robust enough to dissect the specific effects mediated by IFN‐λ3 vs. IFN‐λ4, due to strong LD between the functional variants that regulate the expression of the two genes (Figure 1). Nonetheless, the low‐activity S70 variant gives us an opportunity to test whether IFN‐λ4 is behind an association (Gadalla et al., 2020; Terczyńska‐Dyla et al., 2014). As we have shown in Figure 4 (and Table S5), except for ALP levels, we do not see a significant association involving the S70 variant carriers. Even though, due to limited sample numbers, we cannot dissect out the causal nature of variants controlling either of the genes being responsible for the phenotypes, it appears that IFN‐λ3 has more role to play than IFN‐λ4 in the phenotypes we have tested.

Even though the phenotypes have no direct relationship with any immune responses to pathogens and since we selected participants who were also not affected by any inflammatory disorders, it is surprising to see that the genetic variants are showing extensive cross‐phenotype associations in our cohort. We saw similar results from the PheWAS data in the UK biobank. IFNs in general are central molecules in innate immunity to viruses and tumors in higher organisms (Fuertes et al., 2013; McNab et al., 2015). They could have pleiotropic effects influencing several phenotypes, due to their immunomodulatory functions (De et al., 2021, 2022). But their effects on several non‐immune phenotypes in our study, and supported by PheWAS data from the GBE, raise the possibility of widespread pleiotropy involving IFNs in general and lambda IFNs in particular in human biology. Further, there seems to be a strong gender effect on the associations that we see in our study; this agrees with several previous studies that have documented a gender effect in association of *IFNL* genetic variants with several diseases (Astudillo et al., 2020; Chinnaswamy et al., 2017; Eslam et al., 2015).Cross‐phenotype association is widespread in human complex trait genetics (Solovieff et al., 2013). Moreover, not all cross‐phenotype associations could result from true pleiotropy (Tyler et al., 2016). Discovering pleiotropic effects from GWAS‐identified genetic variants using PheWAS is an active area of research (Hackinger & Zeggini, 2017; Lumsden et al., 2020). However, such studies run the risk of type I error due to multiple testing problems (Pendergrass et al., 2011, 2013; Tyler et al., 2016). Performing conservative multiple testing corrections like the Bonferroni may not always be necessary (Pendergrass et al., 2013) and alternate approaches like choosing those variants that are known to have pleiotropic effects in PheWAS may be the way forward (Hackinger & Zeggini, 2017; Lumsden et al., 2020; Pendergrass et al., 2013). We have not applied any multiple testing correction in our results (except for the results shown in Figure 2). However, we believe that our results are not due to spurious association between genotypes and phenotypes, as IFNs are known to have pleiotropic effects (Borden, 1992), and we see a gender‐specific association with multiple phenotypes in our study, giving a strong biological basis for our observations. Moreover, we see similar multiple cross‐phenotype associations in another population (Figure 5).

Even though we did not have any data to test for genomic inflation, we do not foresee any population stratification in our cohort that may have led to confounding, and therefore, spurious association. This is because the larger combined cohort does not show any association with the genetic variants, suggesting that there is very low chance of population stratification being a potential confounder in our cohort. The association under either dominant or recessive models or the one based on IFN‐λ4 copy number and activity status showed a strong gender‐dependence which argues against any potential structure in the population, that, if present would have shown up roughly equally between the genders. Therefore, we conclude that the novel associations that we report here for *IFNL* genetic variants could be real and the fact that they had better effect sizes than many of the top genes/SNPs for the two phenotypes in the GBE (Figure 6) argues for further research into the gender‐dependent effect of *IFNL* genetic variants in human biology.

The omni‐genic model which postulates that every gene could potentially be contributing to every disease (and probably to health) phenotype is the currently accepted trend in complex trait genetics (Boyle et al., 2017; Mathieson, 2021). In this context, our results could reinforce this model wherein there could be several genes that may be interacting with each other to determine a phenotype. Further studies are needed to increase the gamut of genes that contribute significantly to each phenotype and to understand how they interact collectively among each other. Further research is also needed to understand the different pleiotropic effects of IFN‐λs.

## AUTHOR CONTRIBUTIONS

The study was designed by Sreedhar Chinnaswamy; Debarati Guha Roy collected human samples, genotyped them, analyzed data, visualized the results; Lucky Singh and Himanshu K. Chaturvedi performed data analysis; Sreedhar Chinnaswamy acquired funding, supervised the study, and wrote the manuscript; all authors edited and approved the final draft.

## FUNDING INFORMATION

The author is the recipient of the DBT/Wellcome Trust India Alliance Fellowship (grant number IA/I/17/1/503122).

## CONFLICT OF INTEREST STATEMENT

The authors declare no conflict of interest.

## ETHICS STATEMENT

The study was approved by the National Institute of Biomedical Genomics committee for ethics on human studies (certificate No. NIBMG/2022/1/0018).

## Supporting information


Table S1.

Table S2.

Table S3.

Table S4.

Table S5.

## Data Availability

The data that support the findings of this study are available from the corresponding author upon reasonable request.

## References

[mgg32292-bib-0001] Adam, D. (2019). The gene‐based hack that is revolutionizing epidemiology. Nature, 576(7786), 196–199. 10.1038/d41586-019-03754-3 31822846

[mgg32292-bib-0002] Astudillo, P. , Angulo, J. , Pino, K. , de Carvalho, J. B. , de Morais, G. L. , Perez, S. , de Vasconcelos, A. T. , Ferrés, M. , & López‐Lastra, M. (2020). Correlation between female sex, IL28B genotype, and the clinical severity of bronchiolitis in pediatric patients. Pediatric Research, 87(4), 785–795. 10.1038/s41390-019-0623-1 31645053 PMC7086532

[mgg32292-bib-0003] Bhushan, A. , Ghosh, S. , Bhattacharjee, S. , & Chinnaswamy, S. (2017). Confounding by single nucleotide polymorphism rs117648444 (P70S) affects the association of interferon lambda locus variants with response to interferon‐α‐ribavirin therapy in patients with chronic genotype 3 hepatitis C virus infection. Journal of Interferon & Cytokine Research, 37(8), 369–382. 10.1089/jir.2017.0002 28727946

[mgg32292-bib-0004] Borden, E. C. (1992). Interferons: pleiotropic cellular modulators. Clinical Immunology and Immunopathology, 62(1 Pt 2), S18–S24. 10.1016/0090-1229(92)90037-o 1370263

[mgg32292-bib-0005] Boyle, E. A. , Li, Y. I. , & Pritchard, J. K. (2017). An expanded view of complex traits: From polygenic to Omnigenic. Cell, 169(7), 1177–1186. 10.1016/j.cell.2017.05.038 28622505 PMC5536862

[mgg32292-bib-0006] Chinnaswamy, S. (2016). Gene–disease association with human IFNL locus polymorphisms extends beyond hepatitis C virus infections. Genes & Immunity, 17(5), 265–275. 10.1038/gene.2016.24 27278127 PMC7091887

[mgg32292-bib-0007] Chinnaswamy, S. , Chatterjee, S. , Boopathi, R. , Mukherjee, S. , Bhattacharjee, S. , & Kundu, T. K. (2013). A single nucleotide polymorphism associated with hepatitis C virus infections located in the distal region of the IL28B promoter influences NF‐κB‐mediated gene transcription. PLoS One, 8(10), e75495. 10.1371/journal.pone.0075495 24116050 PMC3792970

[mgg32292-bib-0008] Chinnaswamy, S. , Wardzynska, A. , Pawelczyk, M. , Makowska, J. , Skaaby, T. , & Kowalski, M. L. (2017). A functional IFN‐λ4‐generating DNA polymorphism could protect older asthmatic women from aeroallergen sensitization and associate with clinical features of asthma. Scientific Reports, 7(1), 10500. 10.1038/s41598-017-10467-y 28874741 PMC5585370

[mgg32292-bib-0009] De, M. , Bhushan, A. , & Chinnaswamy, S. (2021). Monocytes differentiated into macrophages and dendritic cells in the presence of human IFN‐λ3 or IFN‐λ4 show distinct phenotypes. Journal of Leukocyte Biology, 110(2), 357–374. 10.1002/JLB.3A0120-001RRR 33205487 PMC7611425

[mgg32292-bib-0010] De, M. , Bhushan, A. , Grubbe, W. S. , Roy, S. , Mendoza, J. L. , & Chinnaswamy, S. (2022). Distinct molecular phenotypes involving several human diseases are induced by IFN‐λ3 and IFN‐λ4 in monocyte‐derived macrophages. Genes and Immunity, 23(2), 73–84. 10.1038/s41435-022-00164-w 35115664 PMC9042695

[mgg32292-bib-0011] Eslam, M. , Hashem, A. M. , Leung, R. , Romero‐Gomez, M. , Berg, T. , Dore, G. J. , Chan, H. L. , Irving, W. L. , Sheridan, D. , Abate, M. L. , Adams, L. A. , & Ahlenstiel, G. (2015). Interferon‐λ rs12979860 genotype and liver fibrosis in viral and non‐viral chronic liver disease. Nature Communications, 6(1), 6422. 10.1038/ncomms7422 PMC4366528 25740255

[mgg32292-bib-0012] Fuertes, M. B. , Woo, S. R. , Burnett, B. , Fu, Y. X. , & Gajewski, T. F. (2013). Type I interferon response and innate immune sensing of cancer. Trends Immunol., 34(2), 67–73. 10.1016/j.it.2012.10.004 23122052 PMC3565059

[mgg32292-bib-0013] Gadalla, S. M. , Wang, Y. , Wang, T. , Onabajo, O. O. , Banday, A. R. , Obajemu, A. , Karaesman, E. , Sucheston‐Campbell, L. , Hahn, T. , Sees, J. A. , Spellman, S. R. , Lee, S. J. , Katki, H. A. , & Prokunina‐Olsson, L. (2020). Association of donor IFNL4 genotype and non‐relapse mortality after unrelated donor myeloablative haematopoietic stem‐cell transplantation for acute leukaemia: A retrospective cohort study. Lancet Haematol, 7(10), e715–e723. 10.1016/S2352-3026(20)30294-5 Erratum in: Lancet Haematol. 2020 Nov;7(11):e785. PMID: 32976751; PMCID: PMC7735535. 32976751 PMC7735535

[mgg32292-bib-0014] Ge, D. , Fellay, J. , Thompson, A. J. , Simon, J. S. , Shianna, K. V. , Urban, T. J. , Heinzen, E. L. , Qiu, P. , Bertelsen, A. H. , Muir, A. J. , Sulkowski, M. , & Goldstein, D. B. (2009). Genetic variation in IL28B predicts hepatitis C treatment‐induced viral clearance. Nature, 461(7262), 399–401. 10.1038/nature08309 19684573

[mgg32292-bib-0015] Ghasemi, A. , & Zahediasl, S. (2012). Normality tests for statistical analysis: A guide for non‐statisticians. International Journal of Endocrinology and Metabolism, 10(2), 486–489. 10.5812/ijem.3505 23843808 PMC3693611

[mgg32292-bib-0016] Hackinger, S. , & Zeggini, E. (2017). Statistical methods to detect pleiotropy in human complex traits. Open Biology, 7(11), 170125. 10.1098/rsob.170125 29093210 PMC5717338

[mgg32292-bib-0017] Hamming, O. J. , Terczyńska‐Dyla, E. , Vieyres, G. , Dijkman, R. , Jørgensen, S. E. , Akhtar, H. , Siupka, P. , Pietschmann, T. , Thiel, V. , & Hartmann, R. (2013). Interferon lambda 4 signals via the IFNλ receptor to regulate antiviral activity against HCV and coronaviruses. The EMBO Journal, 32(23), 3055–3065. 10.1038/emboj.2013.232 24169568 PMC3844954

[mgg32292-bib-0018] Kotenko, S. V. , Gallagher, G. , Baurin, V. V. , Lewis‐Antes, A. , Shen, M. , Shah, N. K. , Langer, J. A. , Sheikh, F. , Dickensheets, H. , & Donnelly, R. P. (2003). IFN‐λs mediate antiviral protection through a distinct class II cytokine receptor complex. Nature Immunology, 4(1), 69–77. 10.1038/ni875 12483210

[mgg32292-bib-0019] Lauber, C. , Vieyres, G. , Terczyńska‐Dyla, E. , Dijkman, R. , Gad, H. H. , Akhtar, H. , Geffers, R. , Vondran, F. W. , Thiel, V. , Kaderali, L. , Pietschmann, T. , & Hartmann, R. (2015). Transcriptome analysis reveals a classical interferon signature induced by IFNλ4 in human primary cells. Genes & Immunity, 16(6), 414–421. 10.1038/gene.2015.23 26066369 PMC7308733

[mgg32292-bib-0020] Lumsden, A. L. , Mulugeta, A. , Zhou, A. , & Hyppönen, E. (2020). Apolipoprotein E (APOE) genotype‐associated disease risks: A phenome‐wide, registry‐based, case‐control study utilising the UK biobank. EBioMedicine., 59, 102954. 10.1016/j.ebiom.2020.102954 32818802 PMC7452404

[mgg32292-bib-0021] Manry, J. , Laval, G. , Patin, E. , Fornarino, S. , Itan, Y. , Fumagalli, M. , Sironi, M. , Tichit, M. , Bouchier, C. , Casanova, J. L. , Barreiro, L. B. , & Quintana‐Murci, L. (2011). Evolutionary genetic dissection of human interferons. Journal of Experimental Medicine, 208(13), 2747–2759. 10.1084/jem.20111680 22162829 PMC3244034

[mgg32292-bib-0022] Mathieson, I. (2021). The omnigenic model and polygenic prediction of complex traits. Am J Hum Genet., 108(9), 1558–1563. 10.1016/j.ajhg.2021.07.003 34331855 PMC8456163

[mgg32292-bib-0023] Matic, S. , Milovanovic, D. , Mijailovic, Z. , Djurdjevic, P. , Sazdanovic, P. , Stefanovic, S. , Todorovic, D. , Popovic, S. , Vitosevic, K. , Vukicevic, V. , Vukic, M. , Vukovic, N. , Milivojevic, N. , Zivanovic, M. , Jakovljevic, V. , Filipovic, N. , Baskic, D. , & Djordjevic, N. (2023). IFNL3/4 polymorphisms as a two‐edged sword: An association with COVID‐19 outcome. Journal of Medical Virology, 95(2), e28506. 10.1002/jmv.28506 36655749

[mgg32292-bib-0024] McFarland, A. P. , Horner, S. M. , Jarret, A. , Joslyn, R. C. , Bindewald, E. , Shapiro, B. A. , Delker, D. A. , Hagedorn, C. H. , Carrington, M. , Gale, M., Jr. , & Savan, R. (2014). The favorable IFNL3 genotype escapes mRNA decay mediated by AU‐rich elements and hepatitis C virus–induced microRNAs. Nature Immunology, 15(1), 72–79. 10.1038/ni.2758 24241692 PMC4183367

[mgg32292-bib-0025] McInnes, G. , Tanigawa, Y. , DeBoever, C. , Lavertu, A. , Olivieri, J. E. , Aguirre, M. , & Rivas, M. A. (2019). Global biobank engine: Enabling genotype‐phenotype browsing for biobank summary statistics. Bioinformatics, 35(14), 2495–2497. 10.1093/bioinformatics/bty999 30520965 PMC6612820

[mgg32292-bib-0026] McNab, F. , Mayer‐Barber, K. , Sher, A. , Wack, A. , & O'Garra, A. (2015). Type I interferons in infectious disease. Nature Reviews Immunology, 15, 87–103. 10.1038/nri3787 PMC7162685 25614319

[mgg32292-bib-0027] Obajemu, A. A. , Rao, N. , Dilley, K. A. , Vargas, J. M. , Sheikh, F. , Donnelly, R. P. , Shabman, R. S. , Meissner, E. G. , Prokunina‐Olsson, L. , & Onabajo, O. O. (2017). IFN‐λ4 attenuates antiviral responses by enhancing negative regulation of IFN signaling. The Journal of Immunology, 199(11), 3808–3820. 10.4049/jimmunol.1700807 29070670 PMC5698113

[mgg32292-bib-0028] Pendergrass, S. A. , Brown‐Gentry, K. , Dudek, S. , Frase, A. , Torstenson, E. S. , Goodloe, R. , Ambite, J. L. , Avery, C. L. , Buyske, S. , Bůžková, P. , Deelman, E. , Fesinmeyer, M. D. , Haiman, C. A. , Heiss, G. , Hindorff, L. A. , Hsu, C. N. , Jackson, R. D. , Kooperberg, C. , Le Marchand, L. , … Ritchie, M. D. (2013). Phenome‐wide association study (PheWAS) for detection of pleiotropy within the population architecture using genomics and epidemiology (PAGE) network. PLoS Genetics, 9(1), e1003087. 10.1371/journal.pgen.1003087 23382687 PMC3561060

[mgg32292-bib-0029] Pendergrass, S. A. , Brown‐Gentry, K. , Dudek, S. M. , Torstenson, E. S. , Ambite, J. L. , Avery, C. L. , Buyske, S. , Cai, C. , Fesinmeyer, M. D. , Haiman, C. , Heiss, G. , Hindorff, L. A. , Hsu, C. N. , Jackson, R. D. , Kooperberg, C. , Le Marchand, L. , Lin, Y. , Matise, T. C. , Moreland, L. , … Ritchie, M. D. (2011). The use of phenome‐wide association studies (PheWAS) for exploration of novel genotype‐phenotype relationships and pleiotropy discovery. Genetic Epidemiology, 35(5), 410–422. 10.1002/gepi.20589 21594894 PMC3116446

[mgg32292-bib-0031] Prokunina‐Olsson, L. (2019). Genetics of the human interferon lambda region. Journal of Interferon & Cytokine Research, 39(10), 599–608. 10.1089/jir.2019.0043 31070498 PMC6767866

[mgg32292-bib-0032] Prokunina‐Olsson, L. , Muchmore, B. , Tang, W. , Pfeiffer, R. M. , Park, H. , Dickensheets, H. , Hergott, D. , Porter‐Gill, P. , Mumy, A. , Kohaar, I. , Chen, S. , & O'Brien, T. R. (2013). A variant upstream of IFNL3 (IL28B) creating a new interferon gene IFNL4 is associated with impaired clearance of hepatitis C virus. Nature Genetics, 45(2), 164–171. 10.1038/ng.2521 23291588 PMC3793390

[mgg32292-bib-0033] Purcell, S. , Neale, B. , Todd‐Brown, K. , Thomas, L. , Ferreira, M. A. R. , Bender, D. , Maller, J. , Sklar, P. , de Bakker, P. I. W. , Daly, M. J. , & Sham, P. C. (2007). PLINK: A tool set for whole‐genome association and population‐based linkage analyses. American Journal of Human Genetics, 81(3), 559–575. 17701901 10.1086/519795 PMC1950838

[mgg32292-bib-0034] Roy, S. , Roy, D. G. , Bhushan, A. , Bharatiya, S. , & Chinnaswamy, S. (2021). Functional genetic variants of the IFN‐λ3 (IL28B) gene and transcription factor interactions on its promoter. Cytokine, 142, 155491. 10.1016/j.cyto.2021.155491 33725487 PMC7611124

[mgg32292-bib-0035] RStudio Team . (2021). RStudio: Integrated development environment for R. RStudio, PBC http://www.rstudio.com/

[mgg32292-bib-0036] Sheppard, P. , Kindsvogel, W. , Xu, W. , Henderson, K. , Schlutsmeyer, S. , Whitmore, T. E. , Kuestner, R. , Garrigues, U. , Birks, C. , Roraback, J. , Ostrander, C. , & Klucher, K. M. (2003). IL‐28, IL‐29 and their class II cytokine receptor IL‐28R. Nature Immunology, 4(1), 63–68. 10.1038/ni873 12469119

[mgg32292-bib-0037] Solovieff, N. , Cotsapas, C. , Lee, P. H. , Purcell, S. M. , & Smoller, J. W. (2013). Pleiotropy in complex traits: Challenges and strategies. Nature Reviews. Genetics, 14(7), 483–495. 10.1038/nrg3461 PMC4104202 23752797

[mgg32292-bib-0038] Suppiah, V. , Moldovan, M. , Ahlenstiel, G. , Berg, T. , Weltman, M. , Abate, M. L. , Bassendine, M. , Spengler, U. , Dore, G. J. , Powell, E. , Riordan, S. , & Hepatitis C Study . (2009). IL28B is associated with response to chronic hepatitis C interferon‐α and ribavirin therapy. Nature Genetics, 41(10), 1100–1104. 10.1038/ng.447 19749758

[mgg32292-bib-0039] Syedbasha, M. , & Egli, A. (2017). Interferon lambda: Modulating immunity in infectious diseases. Frontiers in Immunology, 28(8), 119. 10.3389/fimmu.2017.00119 PMC5328987 28293236

[mgg32292-bib-0040] Tanaka, Y. , Nishida, N. , Sugiyama, M. , Kurosaki, M. , Matsuura, K. , Sakamoto, N. , Nakagawa, M. , Korenaga, M. , Hino, K. , Hige, S. , Ito, Y. , & Mizokami, M. (2009). Genome‐wide association of IL28B with response to pegylated interferon‐α and ribavirin therapy for chronic hepatitis C. Nature Genetics, 41(10), 1105–1109. 10.1038/ng.449 19749757

[mgg32292-bib-0041] Terczyńska‐Dyla, E. , Bibert, S. , Duong, F. H. , Krol, I. , Jørgensen, S. , Collinet, E. , Kutalik, Z. , Aubert, V. , Cerny, A. , Kaiser, L. , Malinverni, R. , & Hartmann, R. (2014). Reduced IFNλ4 activity is associated with improved HCV clearance and reduced expression of interferon‐stimulated genes. Nature Communications, 5(1), 5699. 10.1038/ncomms6699 25534433

[mgg32292-bib-0042] Tyler, A. L. , Crawford, D. C. , & Pendergrass, S. A. (2016). The detection and characterization of pleiotropy: Discovery, progress, and promise. Briefings in Bioinformatics, 17(1), 13–22. 10.1093/bib/bbv050 26223525

