# Supplementary material for: Gender‐dependent multiple cross‐phenotype association of interferon lambda genetic variants with peripheral blood profiles in healthy individuals
Source: Mol Genet Genomic Med. 2023 Oct 5;12(1):e2292. doi: 10.1002/mgg3.2292 (PMC10767428; doi:10.1002/mgg3.2292)
Supplement: Supplementary file 1 [file MGG3-12-e2292-s001.docx]

**Supplementary Table2**: Comparison of blood profiles between males and females. 

| **Sl. No.** | **Phenotype** | **Median-Male** | **Median-Female** | **p-value of Wilcoxon test between medians** | **Rank according to Wilcoxon test p-values*** | **Differences between males and females, expected: Y/N** |
| --- | --- | --- | --- | --- | --- | --- |
| 1 | Hemoglobin | 14.96 | 12.16 | 6.76E-69 | 1 | Y |
| 2 | Total RBC count | 5.03 | 4.38 | 7.86E-50 | 2 | Y |
| 3 | Uric Acid | 6.5 | 4.5 | 8.79E-43 | 3 | Y |
| 4 | Creatinine | 0.91 | 0.68 | 1.30E-41 | 4 | Y |
| 5 | ESR | 16 | 25 | 5.08E-25 | 5 | **N** |
| 6 | GGT | 34.15 | 22.25 | 1.75E-16 | 6 | Y |
| 7 | SGPT | 39.05 | 30.2 | 6.14E-15 | 7 | **N** |
| 8 | Bilirubin Indirect | 0.43 | 0.315 | 3.21E-12 | 8 | **N** |
| 9 | Bilirubin Total | 0.72 | 0.55 | 4.04E-12 | 9 | **N** |
| 10 | Albumin | 4.26 | 4.12 | 4.96E-09 | 10 | **N** |
| 11 | Bilirubin Direct | 0.28 | 0.24 | 1.08E-08 | 11 | **N** |
| 12 | HDL-Cholesterol | 46.6 | 52.05 | 2.15E-08 | 12 | **N** |
| 13 | SGOT | 48.45 | 39.85 | 5.49E-07 | 13 | Y |
| 14 | VLDL | 26.94 | 22.08 | 3.39E-06 | 14 | **N** |
| 15 | Triglycerides | 134.6 | 110.95 | 5.31E-06 | 15 | **N** |
| 16 | Globulin | 3.3 | 3.48 | 1.70E-05 | 16 | **N** |
| 17 | Total Platelet count | 185 | 190 | 0.00183 | 17 | **N** |
| 18 | % of Neutrophils | 57.86 | 60 | 0.00318 | 18 | **N** |
| 19 | % of Eosinophils. | 3 | 2.99 | 0.03542 | 20 | **N** |
| 20 | % of Lymphocytes | 35.1 | 35 | 0.05344 | 21 | **N** |
| 21 | % of Monocytes | 2.6 | 2 | 0.0767 | 23 | **N** |
| 22 | Non-HDL Cholesterol | 127.4 | 123.4 | 0.08777 | 24 | **N** |
| 23 | TSH | 2.04 | 2.124 | 0.2996 | 27 | N |
| 24 | % of Basophils | 0 | 0 | 0.44975 | 29 | N |
| 25 | Blood Glucose Fasting | 91.8 | 92.2 | 0.46126 | 30 | N |
| 26 | Total leukocyte count | 7.24 | 7.43 | 0.48254 | 31 | N |
| 27 | ALP | 90 | 93 | 0.48472 | 32 | Y |
| 28 | Total Protein | 7.585 | 7.655 | 0.59669 | 33 | N |
| 29 | Total Cholesterol | 177 | 175.8 | 0.65754 | 34 | N |
| 30 | LDL-Cholesterol | 95.74 | 99.14 | 0.78359 | 35 | N |

The difference between the genders in median values was tested using Wilcoxon test. HDL-cholesterol: High Density Lipoprotein -cholesterol, LDL-cholesterol: Low Density Lipoprotein -cholesterol, VLDL-cholesterol: Very Low-Density Lipoprotein -cholesterol, SGPT: Serum Glutamic Pyruvic Transaminase, SGOT: Serum Glutamic Oxaloacetic Transaminase, ALP: Alkaline Phosphatase, GGT: Gamma Glutamyl Transferase, TSH: Thyroid Stimulating Hormone; Y-Yes; N-No. *The ranking is based on results for 35 phenotypes (only 30 results are shown here; the remaining are absolute counts for neutrophils, eosinophils, lymphocytes, basophils and monocytes).
