# Supplementary material for: Gender‐dependent multiple cross‐phenotype association of interferon lambda genetic variants with peripheral blood profiles in healthy individuals
Source: Mol Genet Genomic Med. 2023 Oct 5;12(1):e2292. doi: 10.1002/mgg3.2292 (PMC10767428; doi:10.1002/mgg3.2292)
Supplement: Supplementary file 2 [file MGG3-12-e2292-s002.docx]

**Suppl. Table 5: Linear regression analysis of five clinical phenotypes (shown in Fig. 4) based on three genotype groupings (No L4= no IFN-L4; P70= P70-1+P70-2 and only S70= only S70-1) instead of four groupings (No IFN-L4, P70-1, P70-2 and S70-1, as detailed in Fig. 4).**

|  |  | Estimates | Std. Error | t-value | Pr(>│t│) |  |
| --- | --- | --- | --- | --- | --- | --- |
| **combined** | No L4 vs P70 | -4.319 | 9.166 | -0.471 | 0.6377 | **Triglycerides** |
|  | No L4 vs S70 | -5.774 | 8.926 | -0.647 | 0.51813 |  |
|  | P70 vs S70 | -6.7 | 17.497 | -0.383 | 0.70216 |  |
|  | No L4 vs P70 | -0.1442 | 1.5731 | -0.092 | 0.92699 | **VLDL** |
|  | No L4 vs S70 | -0.7844 | 1.3976 | -0.561 | 0.574965 |  |
|  | P70 vs S70 | -1.343 | 3.5 | -0.384 | 0.70153 |  |
|  | No L4 vs P70 | -4.91051 | 3.4141 | -1.438 | 0.151 | **ALP** |
|  | No L4 vs S70 | 1.5962 | 3.3877 | 0.471 | 0.638 |  |
|  | P70 vs S70 | 8.393 | 6.297 | 1.333 | 0.184 |  |
| **Males** | No L4 vs P70 | 1.46 | 13.62 | 0.107 | 0.9148 | **Triglycerides** |
|  | No L4 vs S70 | -9.764 | 11.657 | -0.838 | 0.4034 |  |
|  | P70 vs S70 | -20.37 | 32.11 | -0.634 | 0.527 |  |
|  | No L4 vs P70 | 0.2878 | 2.7246 | 0.106 | 0.916 | **VLDL** |
|  | No L4 vs S70 | -1.96 | 2.331 | -0.841 | 0.401433 |  |
|  | P70 vs S70 | -4.085 | 6.422 | -0.636 | 0.526 |  |
|  | No L4 vs P70 | -6.462 | 4.985 | -1.296 | 0.196 | **ALP** |
|  | No L4 vs S70 | 2.458 | 5.169 | 0.476 | 0.635 |  |
|  | P70 vs S70 | 11.484 | 8.845 | 1.298 | 0.196877 |  |
|  | No L4 vs P70 | -0.5973 | 0.2106 | -2.836 | **0.00493** | **Monocyte %** |
|  | No L4 vs S70 | -0.2954 | 0.2361 | -1.251 | 0.212 |  |
|  | P70 vs S70 | 0.008643 | 0.254531 | 0.034 | 0.972972 |  |
|  | No L4 vs P70 | 0.06198 | 0.17806 | 0.348 | 0.728 | **Uric acid** |
|  | No L4 vs S70 | 0.02997 | 0.17395 | 0.172 | 0.863 |  |
|  | P70 vs S70 | 0.002294 | 0.3858 | 0.006 | 0.995 |  |

The groupings tested are: 

**No L4**- No IFN-L4 (TT/TT at rs368234815 and G/G at rs117648444); 

**P70**-at least one copy of P70 (TT/ΔG at rs368234815 and G/G at rs117648444 with ΔG/ΔG at rs368234815 and G/G + G/A at rs117648444); 

**S70**-only one copy of S70 (TT/ ΔG at rs368234815 and G/A at rs117648444).
