# Supplementary material for: Gender‐dependent multiple cross‐phenotype association of interferon lambda genetic variants with peripheral blood profiles in healthy individuals
Source: Mol Genet Genomic Med. 2023 Oct 5;12(1):e2292. doi: 10.1002/mgg3.2292 (PMC10767428; doi:10.1002/mgg3.2292)
Supplement: Supplementary file 3 [file MGG3-12-e2292-s003.docx]

Sup Table 3: List of demographic/lifestyle variables used in the study to test for confounding.

| **Sl. No.** | **Demographic Variables** | **N (%)** |
| --- | --- | --- |
| **1** | **Age in years** |  |
|  | 29 and below | 224 (40.57) |
|  | 30-44 | 207 (37.5) |
|  | 45 and above | 121 (21.92) |
| **2** | **Type of House** |  |
|  | Pukka | 395 (71.55) |
|  | Semi Pukka | 105 (19.02) |
|  | Kaccha | 52 (9.42) |
| **3** | **place of House** |  |
|  | Urban | 135 (24.45) |
|  | Rural | 417 (75.54) |
| **4** | **Food habit** |  |
|  | Vegetarian | 110 (19.92) |
|  | Non-vegetarian | 442 (80.07) |
| **5** | **Alcohol consumption** |  |
|  | Yes | 179 (32.42) |
|  | No | 373 (67.57) |
| **6** | **Smoking** |  |
|  | Yes | 150 (27.17) |
|  | No | 402 (72.82) |
| **7** | **Any other Addiction** |  |
|  | Yes | 47 (8.51) |
|  | No | 505 (91.48) |
| **8** | **Occupation** |  |
|  | Agriculture | 6 (1.08) |
|  | Business | 175 (31.70) |
|  | professional | 70 (12.68) |
|  | Student | 115 (20.83) |
|  | Housewife | 166 (30.07) |
|  | others | 20 (3.62) |
| **9** | **Annual income of family** |  |
|  | up to Rs 25000 | 29 (5.25) |
|  | Rs 25000 to 1.5 lacks | 410 (74.27) |
|  | Rs 1.5 lacs to 5 lacks | 86 (15.57) |
|  | more than Rs 5 lacks | 27 (4.89) |
| **10** | **Source of drinking water in the household** |  |
|  | Tap water | 420 (76.08) |
|  | hand Pump | 52 (9.42) |
|  | Tube well | 80 (14.49) |
| **11** | **Drink purified water** |  |
|  | Yes | 245 (44.38) |
|  | No | 307 (55.61) |
