# Supplementary material for: Gender‐dependent multiple cross‐phenotype association of interferon lambda genetic variants with peripheral blood profiles in healthy individuals
Source: Mol Genet Genomic Med. 2023 Oct 5;12(1):e2292. doi: 10.1002/mgg3.2292 (PMC10767428; doi:10.1002/mgg3.2292)
Supplement: Supplementary file 4 [file MGG3-12-e2292-s004.docx]

Supplementary Table 4: The effect of *IFNL3/4* SNP on blood phenotypes adjusted for age and sex or age only. Multiple linear regression was performed. Only Genetic variants corresponding p-values are shown in this table. p-value<0.05 was considered as significant and highlighted in bold.

| **Parameter** | **Status** | **Genetic Variants/model** | **Adjusted for** | **p-value** |
| --- | --- | --- | --- | --- |
| % Monocytes | Male | rs12979860 Dominant | Age | **0.00168** |
| % Monocytes | Male | No-IFNL4 vs P70-1 | Age | **0.0132** |
|  |  |  |  |  |
| SGOT | Male | rs28416813 Dominant | Age | **0.00741** |
|  |  |  |  |  |
| Uric Acid | Male | rs12979860 Recessive | Age | **0.0163** |
| Uric Acid | Male | No-IFNL4 vs P70-2 | Age | **0.037** |
| Uric Acid | Male | P70-1 vs P70-2 | Age | **0.0186** |
|  |  |  |  |  |
| Globulin | Male | rs12979860 Recessive | Age | 0.0557 |
|  |  |  |  |  |
| Triglycerides | Combined | No-IFNL4 vs P70-2 | Age and Gender | **0.04123** |
| Triglycerides | Combined | P70-1 vs P70-2 | Age and Gender | **0.02145** |
| Triglycerides | Combined | P70-2 vs S70 | Age and Gender | 0.0839 |
| Triglycerides | Male | No-IFNL4 vs P70-2 | Age | **0.01595** |
| Triglycerides | Male | P70-1 vs P70-2 | Age | **0.0307** |
|  |  |  |  |  |
| VLDL | Combined | No-IFNL4 vs P70-2 | Age and Gender | **0.017771** |
| VLDL | Combined | P70-1 vs P70-2 | Age and Gender | **0.02136** |
| VLDL | Combined | P70-2 vs S70 | Age and Gender | 0.0839 |
| VLDL | Male | No-IFNL4 vs P70-2 | Age | **0.01587** |
| VLDL | Male | P70-1 vs P70-2 | Age | **0.0307** |
|  |  |  |  |  |
| ALP | Combined | P70-1 vs S70 | Age and Gender | **0.0341** |
| ALP | Male | P70-1 vs S70 | Age | **0.00219** |
|  |  |  |  |  |
| Albumin | Female | rs12979860 Recessive | Age | **0.0168** |
|  |  |  |  |  |
| % Eosinophil | Combined | P70-1 vs S70 | Age and Gender | 0.676773 |
| % Eosinophil | Female | P70-1 vs S70 | Age | 0.6418 |
|  |  |  |  |  |
| Creatinine | Female | P70-2 vs S70 | Age | 0.0625 |
| Platelet count | Female | rs12979860 Recessive | Age | 0.0964 |
|  |  |  |  |  |

VLDL-cholesterol: Very Low-Density Lipoprotein -cholesterol, SGOT: Serum Glutamic Oxaloacetic Transaminase, ALP: Alkaline Phosphatase.
