# Supplementary material for: Gender‐dependent multiple cross‐phenotype association of interferon lambda genetic variants with peripheral blood profiles in healthy individuals
Source: Mol Genet Genomic Med. 2023 Oct 5;12(1):e2292. doi: 10.1002/mgg3.2292 (PMC10767428; doi:10.1002/mgg3.2292)
Supplement: Supplementary file 5 [file MGG3-12-e2292-s005.docx]

**Supplementary Table1**: Peripheral blood phenotypes of healthy participants in the study. The normal values with ranges were provided by the lab that carried out the tests for the study. Any deviation from the reference ranges is highlighted in bold.

|  |  | **Combined** |  |  | **Male** |  |  |  |  |  | **Female** |  |  |  |  |  |
| --- | --- | --- | --- | --- | --- | --- | --- | --- | --- | --- | --- | --- | --- | --- | --- | --- |
| **Parameters** | **Normal range** | **N** | **Mean** | **Median** | **Normal range** | **N** | **Mean** | **Status** | **Median** | **Status** | **Normal range** | **N** | **Mean** | **Status** | **Median** | **Status** |
| Hemoglobin | Male: 13-17 g/dl, female: 12-15 g/dl | 550 | 13.439 | 13.4 | Male: 13-17 g/dl | 283 | 14.8252 | Normal | 14.96 | Normal | Female: 12-15 g/dl | 267 | **11.9697** | Low | 12.16 | Normal |
| RBC count | Male: 4.5-5.5 millions/mm^3^, female: 3.8-4.8 millions/mm^3^ | 550 | 4.73456 | 4.7 | Male: 4.5-5.5 millions/mm^3 | 283 | 5.0688 | Normal | 5.03 | Normal | Female: 3.8-4.8 millions/mm^3 | 267 | 4.3803 | Normal | 4.38 | Normal |
| Platelet count | 150-410 10^3/µl | 550 | 205.942 | 185 | 150-410 10^3/µl | 283 | 194.29 | Normal | 185 | Normal | 150-410 10^3/µl | 267 | 218.292 | Normal | 190 | Normal |
| Total leukocyte count | 4-10 10^3/µl | 550 | 7.4928 | 7.295 | 4-10 10^3/µl | 283 | 7.46007 | Normal | 7.24 | Normal | 4-10 10^3/µl | 267 | 7.52749 | Normal | 7.43 | Normal |
| % of Neutrophils | 40-80 | 550 | 59.1703 | 59 | 40-80 | 283 | 58.0399 | Normal | 57.86 | Normal | 40-80 | 267 | 60.3685 | Normal | 60 | Normal |
| % of Lymphocytes | 20-40 | 550 | 34.1656 | 35 | 20-40 | 283 | 34.9492 | Normal | 35.1 | Normal | 20-40 | 267 | 33.335 | Normal | 35 | Normal |
| % of Eosinophils | 1-6 | 550 | 3.86985 | 3 | 1-6 | 283 | 4.02883 | Normal | 3 | Normal | 1-6 | 267 | 3.70135 | Normal | 2.99 | Normal |
| % of Monocytes | 2-10 | 550 | 2.77093 | 2 | 2-10 | 283 | 2.94078 | Normal | 2.6 | Normal | 2-10 | 267 | 2.5909 | Normal | 2 | Normal |
| % of Basophils | 0-1 | 550 | 0.0286 | 0 | 0-1 | 283 | 0.03392 | Normal | 0 | Normal | 0-1 | 267 | 0.02296 | Normal | 0 | Normal |
| Erythrocyte sedimentation rate | 0-15 mm in 1st hour | 524 | 23.4981 | 20 | 0-15 mm in 1st hour | 265 | **18.5321** | High | **16** | High | 0-15 mm in 1st hour | 259 | **28.5792** | High | **25** | High |
| Total Cholesterol | <200mg/dL | 551 | 178.991 | 176 | <200mg/dL | 283 | 180.139 | Normal | 177 | Normal | <200mg/dL | 268 | 177.778 | Normal | 175.8 | Normal |
| Triglycerides | <150 mg/dL | 551 | 145.845 | 122.6 | <150 mg/dL | 283 | **161.771** | High | 134.6 | Normal | <150 mg/dL | 268 | 129.028 | Normal | 110.95 | Normal |
| HDL Cholesterol | >40 mg/dL | 551 | 50.6145 | 49.4 | >40 mg/dL | 283 | 48.5233 | Normal | 46.6 | Normal | >40 mg/dL | 268 | 52.8228 | Normal | 52.05 | Normal |
| Non-HDL Cholesterol | <130 mg/dL | 545 | 128.369 | 126.7 | <130 mg/dL | 280 | **131.75** | High | 127.4 | Normal | <130 mg/dL | 265 | 124.797 | Normal | 123.4 | Normal |
| LDL- Cholesterol | <130 mg/dL | 551 | 99.3975 | 97.7 | <130 mg/dL | 283 | 99.6165 | Normal | 95.74 | Normal | <130 mg/dL | 268 | 99.1663 | Normal | 99.14 | Normal |
| VLDL | 0-45 mg/dL | 550 | 28.7555 | 24.5 | 0-45 mg/dL | 283 | 32.3558 | Normal | 26.94 | Normal | 0-45 mg/dL | 267 | 24.9394 | Normal | 22.08 | Normal |
| Bilirubin Total | 0.1-1.2 mg/dL | 552 | 0.7162 | 0.63 | 0.1-1.2 mg/dL | 284 | 0.82162 | Normal | 0.72 | Normal | 0.1-1.2 mg/dL | 268 | 0.60448 | Normal | 0.55 | Normal |
| Bilirubin Direct | <0.2 mg/dL | 552 | 0.2831 | 0.26 | <0.2 mg/dL | 284 | **0.3169** | High | **0.28** | High | <0.2 mg/dL | 268 | **0.24728** | High | **0.24** | High |
| Bilirubin Indirect | 0.8 mg/dL | 552 | 0.43308 | 0.365 | 0.8 mg/dL | 284 | 0.50468 | Normal | 0.43 | Normal | 0.8 mg/dL | 268 | 0.3572 | Normal | 0.315 | Normal |
| SGOT | Male: 0-40 IU/L, Female: 0-32 IU/L | 552 | 49.7342 | 45.05 | Male: 0-40 IU/L | 284 | **53.8306** | High | **48.45** | High | Female: 0-32 IU/L | 268 | **45.3933** | High | **39.85** | High |
| SGPT | 0-35 U/L | 552 | 41.0991 | 35 | 0-35 U/L | 284 | **48.2317** | High | **39.05** | High | 0-35 U/L | 268 | 33.5407 | Normal | 30.2 | Normal |
| ALP | Male: 53-128 U/L, Female: 42-98 U/L | 552 | 98.6092 | 92 | Male: 53-128 U/L | 284 | 98.8053 | Normal | 90 | Normal | Female: 42-98 U/L | 268 | **98.4015** | High | 93 | Normal |
| GGT | Male: 12-58 U/L, Female: 12-43 U/L | 538 | 36.3086 | 27 | Male: 12-58 U/L | 278 | 44.3313 | Normal | 34.15 | Normal | Female: 12-43 U/L | 260 | 27.7304 | Normal | 22.25 | Normal |
| Total Protein | 6-8.3 g/dl | 552 | 7.71437 | 7.61 | 6-8.3 g/dl | 284 | 7.83007 | Normal | 7.585 | Normal | 6-8.3 g/dl | 268 | 7.59175 | Normal | 7.655 | Normal |
| Albumin | 3.2-5.2 g/dl | 552 | 4.2224 | 4.2 | 3.2-5.2 g/dl | 284 | 4.29544 | Normal | 4.26 | Normal | 3.2-5.2 g/dl | 268 | 4.14526 | Normal | 4.12 | Normal |
| Globulin | 2.3-4.5 g/dl | 552 | 3.36813 | 3.38 | 2.3-4.5 g/dl | 284 | 3.29415 | Normal | 3.3 | Normal | 2.3-4.5 g/dl | 268 | 3.44653 | Normal | 3.48 | Normal |
| Creatinine | Male: 0.9-1.3 mg/dL, Female: 0.6-1.1 mg/dL | 552 | 0.80491 | 0.79 | Male: 0.9-1.3 mg/dL | 284 | **0.89908** | Low | 0.91 | Normal | Female: 0.6-1.1 mg/dL | 268 | 0.70511 | Normal | 0.68 | Normal |
| Blood Glucose Fasting | <100 mg/dL | 552 | 97.9431 | 92 | <100 mg/dL | 284 | 98.4461 | Normal | 91.8 | Normal | <100 mg/dL | 268 | 97.4081 | Normal | 92.2 | Normal |
| Uric Acid | Male: 3.5-7.2 mg/dL, Female: 2.4-6 mg/dL | 550 | 5.58231 | 5.5 | Male: 3.5-7.2 mg/dL | 283 | 6.44774 | Normal | 6.5 | Normal | Female: 2.4-6 mg/dL | 267 | 4.66502 | Normal | 4.5 | Normal |
| TSH | 0.35-5.5 µlU/mL | 551 | 2.90593 | 2.063 | 0.35-5.5 µlU/mL | 283 | 2.88112 | Normal | 2.04 | Normal | 0.35-5.5 µlU/mL | 268 | 2.93213 | Normal | 2.124 | Normal |

	HDL-cholesterol: High Density Lipoprotein -cholesterol, LDL-cholesterol: Low Density Lipoprotein -cholesterol, VLDL-cholesterol: Very Low-Density Lipoprotein -cholesterol, SGPT: Serum Glutamic Pyruvic Transaminase, SGOT: Serum Glutamic Oxaloacetic Transaminase, ALP: Alkaline Phosphatase, GGT: Gamma Glutamyl Transferase, TSH: Thyroid Stimulating Hormone.
